# Microplastic contamination in three environmental compartments of a coastal lagoon in the southern Gulf of Mexico

**DOI:** 10.1007/s10661-024-13156-2

**Published:** 2024-10-04

**Authors:** Mitzi Sánchez-Campos, Guadalupe Ponce-Vélez, Laura Sanvicente-Añorve, Miguel Alatorre-Mendieta

**Affiliations:** 1 Posgrado en Ciencias del Mar y Limnología, Universidad Nacional Autónoma de México, Ciudad de Mexico, Mexico; Av. Universidad 3000, Ciudad Universitaria Coyoacán, C.P. 04510 Ciudad de Mexico, Mexico; 2 Laboratorio de Contaminación Marina, Instituto de Ciencias del Mar y Limnología, Universidad Nacional Autónoma de México, Ciudad de Mexico, Mexico; 3 Laboratorio de Ecología de Sistemas Pelágicos, Instituto de Ciencias del Mar y Limnología, Universidad Nacional Autónoma de México, Ciudad de Mexico, Mexico; 4 Laboratorio de Oceanografía Física, Instituto de Ciencias del Mar y Limnología, Universidad Nacional Autónoma de México, Ciudad de Mexico, Mexico

**Keywords:** Microplastics, Water, Zooplankton, Sediment, Estuary

## Abstract

The Sontecomapan lagoon (Mexico) is a Ramsar site within the Los Tuxtlas Biosphere Reserve, facing the Gulf of Mexico. Although the site has a protected area status, it is vulnerable to microplastic contamination, whose long-term effects are uncertain. This study gives the first approach to the degree of contamination by microplastics in surface waters, zooplankton, and sediments in the lagoon. The samples in these three environmental compartments were collected in June 2018 and analyzed in the laboratory to extract and quantify the microplastics. The microplastics sampled were classified into fibers, fragments, and foams and identified as polyester, acrylic, and rayon, among others. In the surface waters, the mean concentration of microplastics was 7.5 ± 5.3 items/L, which is higher than the values registered in other protected coastal systems, perhaps because of differences in the methods used. Zooplankton, represented by copepods, luciferids, and chaetognaths, showed concentrations of 0.002 ± 0.005, 0.011 ± 0.011, and 0.019 ± 0.016 items/individual, respectively. These values were low compared to systems with high anthropic influence, and the differences between the three kinds of organisms were attributed to their feeding habits. In the sediments, the mean concentration was 8.5 ± 12.5 items/kg, lower than the values registered in sites of high human impact; the maximum value here found (43 items/kg) was recorded in the internal part of a lagoon arm of almost stagnant water. In general, the degree of contamination by microplastics in the lagoon was low; however, their presence indicates a potential risk to the biota.

**Supplementary information:**

The online version contains supplementary material available at 10.1007/s10661-024-13156-2.

## Introduction

Coastal lagoons and estuaries are highly productive ecosystems because of the variety of primary producers, they contain a broad range of habitats serving as nursery grounds for larvae and juvenile organisms, refugia against predators, or permanent habitats for resident species, and they play a key role in the recycling of nutrients (Boynton & Nixon, 2013; Thrush et al., 2013). Coastal lagoons contribute to the regulation of water quality and climate as well as in the protection of coastlines from erosion and flooding; for the coastal human population, they offer food sources, raw materials, and a way of survival, and they also represent areas of great economic, cultural, and recreational interest (Garcés-Ordóñez et al., 2022b; Newton et al., 2018; Thrush et al., 2013). However, despite the great benefits that estuaries and lagoons provide to human populations, the anthropogenic pressure over them is a constant threat to ecosystem health. To protect the ecosystems from human impact, a policy instrument, the Marine Protected Areas (MPAs), was established throughout the world to preserve marine and coastal biodiversity, ecosystem function and services, and cultural heritage (IUCN, 2004; OECD, 2017).

Marine and coastal ecosystems are exposed to multiple sources of contaminants, from which plastics constitute up to 80% of marine debris (IUCN, 2021), with adverse effects on the environment and the great variety of living organisms they harbor (Auta et al., 2017; Taha et al., 2021). Large plastics can be fragmented and become microplastics, whose detrimental impact is enhanced by their lightweight and small size which allows an easy intake by organisms and transfer into aquatic food webs (Sharma et al., 2021). Plastics commonly encountered in the marine environment are made of persistent recalcitrant materials; natural degradation can ultimately occur but at a very slow rate (Andrady, 2015). Due to their persistence and low density, microplastics are widely distributed in the oceans and are viewed as one of the major health threats in both the benthic and pelagic environments (Dharmadasa et al., 2021; Nunes et al., 2023a, 2023b). Because not even MPAs are exempt from the presence of microplastics, recent studies have used large databases of microplastics and/or maps of species distribution to detect vulnerable sites of plastic exposure (Compa et al., 2023; Nunes et al., 2023a, 2023b).

The presence of plastic particles can affect the system’s abiotic properties, such as light penetration (a key factor for primary producers), and sediment characteristics such as enzymatic reduction and microbial diversity (Li et al., 2022; Rodrigues et al., 2018a). Besides, the density of microplastics largely determines their presence in either the sediment or the water column. Particularly, in shallow estuarine systems, the resuspension dynamics of sediments induced by wind and/or waves temporarily contribute to a substantial increase of microplastics in the water column (Brand et al., 2015; Vermeiren et al., 2016). Consequently, the inhabitants of the water column are exposed to those suspended particles. Such is the case of zooplankton, which plays an important role in the energy flow between primary producers and major consumers and exhibits a great variety of feeding methods (Brito et al., 2016; Keister & Bonnet, 2012; Kiørboe, 2011). The ingestion of plastic particles by zooplankton can harm or obstruct the intestinal tract, decrease the ability to ingest and digest food, reduce the growth, fecundity, and reproductive output, increase mortality in early stages, affect the swimming behavior by reducing the travel distance and the swimming velocity, diminish the time and size of settling, and alter the body shape (Botterell et al., 2019; Lee et al., 2013). Besides, microplastics may introduce toxic substances to organisms by releasing the additives used during their manufacturing, or by dissociating the contaminants adsorbed to the microplastic surface (Bridson et al., 2023; Fu et al., 2021). Externally, microplastics can adhere to animal appendages, thereby impeding locomotion, ingestion, mating, and mechanoreception (Cole et al., 2013).

The Mexican coast of the Gulf of Mexico is an extensive plain characterized by dunes, mangrove areas, rivers with diverse discharge volumes, and volcanic rock outcrops. This region is of great socio-economic importance for the country due to the presence of archaeological sites, tourism areas, and a wide variety of fishing resources (Zárate-Lomelí et al., 1999). Of particular interest in the region is the Sontecomapan lagoon, which is part of the Los Tuxtlas Biosphere Reserve (TBR), a protected area included in category VI of sustainable use of natural resources according to the International Union for Conservation of Nature (IUCN). This lagoon was classified as a Ramsar site in 2004 owing to the presence of one of the best-preserved mangroves on the coast of the Gulf of Mexico. In addition to its importance as a zone of conservation of biological diversity, the area harbors several threatened and endemic species and is a resting and refuge site for numerous migratory birds coming from temperate regions of North America (Gómez-Marín, 2003; Secretaría de la Convención de Ramsar, 2010).

The lagoon is located within the municipality of Catemaco and Sontecomapan is the village closest to the lagoon, both within the TBR. The economic activities of Catemaco generate a considerable quantity of plastics and other types of solid wastes which in 2018 reached almost 70 tons per day (INEGI, 2019). The sources of plastics in the area are varied: in the lagoon and adjacent marine zone, local fishing activities can release synthetic fibers from the nets, and, in the terrestrial environment, agriculture and cattle farming use a variety of plastic tools. Furthermore, the quotidian labors of the inhabitants combined with small-scale tourism activities generate plastic waste that eventually reaches the lagoon. Unfortunately, there is no knowledge of the amount of macro- or microplastics in any of the different environments of the lagoon, including inflowing rivers. Indeed, studies in Mexican coastal systems from the Gulf of Mexico concerning microplastic contamination are scarce (Cruz-Salas et al., 2022; Montoya-Melgoza et al., 2024; Sánchez-Hernández et al., 2021).

Ecosystem services provided by the Sontecomapan lagoon to the coastal population are highly important because it is a significant part of the natural capital needed for human well-being (Carmona-Díaz et al., 2004). Therefore, it is crucial to preserve the integrity of the lagoon and its environs to guarantee the sustainable development of coastal human settlements (TEEB, 2010). Despite the efforts of local fishermen to preserve the lagoon, there are no formal strategies addressed to mitigate damage and conserve the ecosystem. An important step in the ecosystem’s conservation is to determine the condition of environmental contaminants (GESAMP, 2001). Thus, given the growing quantity of plastic waste in the region and the ecological and economic significance of the lagoon on the Mexican coast of the Gulf of Mexico, this study aims to assess the degree of microplastic contamination in the Sontecomapan lagoon in different environmental compartments: water, zooplankton, and sediments, key components of aquatic ecosystems that provide a comprehensive view of the levels of contamination. Since in Mexico, there is not yet regulation for plastic waste disposal, this study aims to locate the level of microplastic contamination compared with other sites in the world. We hypothesize that the degree of contamination will be lower than in highly populated areas because the lagoon has a relatively high degree of conservation. Regarding the three environmental compartments, we also expect a higher quantity of microplastics in the sediments because of their role as long-term sinks for microplastics and other contaminants.

## Materials and methods

### Study area

The Sontecomapan lagoon is a tropical coastal system with an area of approximately 8.9 km^2^ (7 km long and 1.5 km wide) and an average depth of 1.5 m, on the coast of the southern Gulf of Mexico. The lagoon has a permanent connection to the ocean through a 137-m-wide channel. The climate is humid tropical with pluvial precipitation varying between 3000 and 4000 mm/year (Aké-Castillo et al., 2006). Mean air temperature ranges from 22 °C in January to 27 °C in July (https://www.windy.com), sunlight period varies between 11 and 13.2 h/day in a year (https://wanderlog.com/weather), and solar radiation varies between 6.75 and 7.68 kWh/m^2^ (https://areas.geofisica.unam.mx/radiacion_solar/datos.html).

**Fig. 1:**
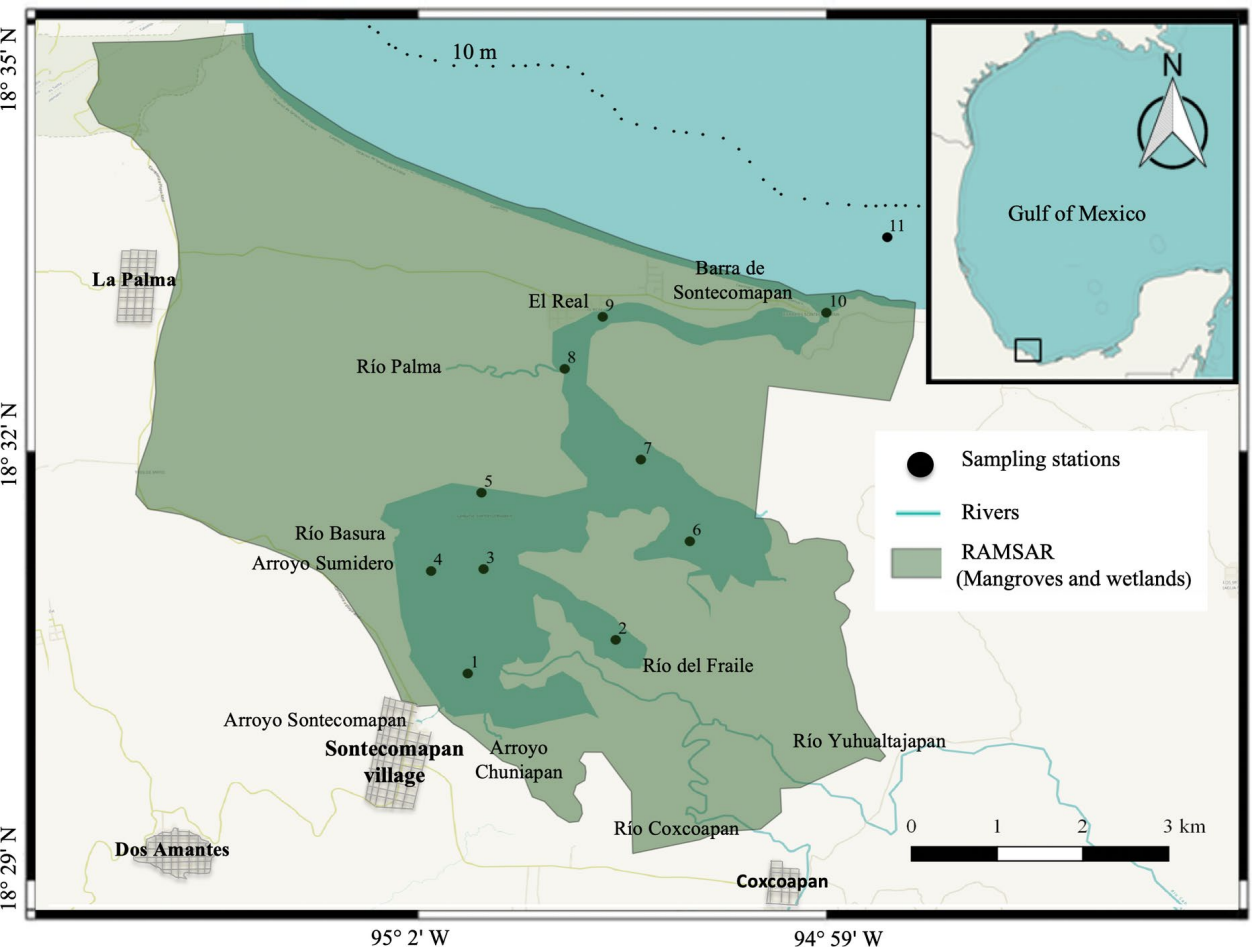
Geographical position of the Sontecomapan lagoon and location of sampling stations

Based on wind and rainfall patterns, three meteorological conditions can be distinguished throughout a year: the “nortes” season lasting from November to February and characterized by strong and cold northern winds and sporadic rainfall; the “dry” season, from March to early June, typified by easterly and southeasterly winds and low pluvial precipitation; and the “rainy” season, lasting from late June to October and characterized by continuous freshwater inflow coming from the rivers and runoff caused by high precipitation (Esquivel-Herrera & Soto-Castor, 2018; Yáñez-Arancibia & Day, 1982); however, interannual variability in the extension of these seasons exists. Most of the time (85%), the tides in the lagoon are on the diurnal type and the amplitude varies between 30 and 60 cm for the neap and spring tides, respectively (https://predmar.cicese.mx/calendarios/). The system has marked seasonal fluctuation in salinity, resulting from the variability in the tides and in the amounts of fresh water from rivers such as Coxcoapan, Yuhualtajapan, Palma, del Fraile, and Basura (Fig. 1). Particularly, on June 19, 2018 (the sampling date), salinity varied between 18.2 psu in the innermost section and 32.5 psu in the mouth area; at the end of the channel (station 8), salinity was 25.8 psu, and to the south (stations 5, 6, and 7), salinity was around 23.5 psu, indicating a wide intrusion of salinity waters. The surface current’s velocity (measured with drifting buoys) was low and varied between almost zero to 0.1 m/s. Based on these data, one could expect that the highest concentration of microplastics in sediments could occur in areas of almost null velocity, due to a higher rate of sedimentation in the water column.

As stated, the largest village adjoining the lagoon is Sontecomapan (2,432 inhabitants) (Gómez-Marín, 2003; INEGI, 2021). The inhabitants of this and other small settlements around the lagoon exert intense pressure on the surrounding vegetation due to the change in land use, whose wastes reach the lagoon system. In 2018, the municipality of Catemaco generated an average of 68,000 kg/day of solid waste, including plastics (INEGI, 2019). The area supports economic activities that could be responsible for the plastic waste, such as the fishery based on artisanal fishing gear, where nylon nets can release synthetic fibers. Main fishing resources include several kinds of fish (seabasses, snappers, croakers, mullets), bivalves (oysters), and crustaceans (white shrimps) (González-Fierro & Ponce-Vélez, 2018). In the terrestrial environment, extensive cattle farming and crop production use plastic, for example, in greenhouses, mulch, irrigation systems, and planters. The small-scale tourism (restaurants, hotels, eco-tourism, baths) activity produces waste that eventually reaches the lagoon or the rivers that connect to the lagoon. Unfortunately, there is no appropriate waste management and, as in other countries, there is still a lack of ecological awareness of the inhabitants around the lagoon.

### Fieldwork

Water, sediment, and zooplankton samples were collected in June 2018 at 11 sampling stations (Fig. 1); these sites were established to cover the entire lagoon body and its different environments: lagoon mouth, freshwater influence areas, “nook” sites, and proximity to docks or human settlements (Fig. 1). The water samples were composite samples; they were recovered at 1 m depth with a 10-L sampler and represent the integration of four subsamples obtained at each sampling site; the composite sample was manually homogenized to obtain a 1-L aliquot for laboratory analysis. Recent studies have established that volumes between 1 and 2 L of water in natural environments represent a common and appropriate sample size for the analysis of microplastics in aquatic ecosystems (Gao et al., 2023; Huang et al., 2021; Idowu et al., 2024). The sediments were sampled (1 kg) with a van Veen grab; samples were composite samples, obtained from three dredge hauls at each sampling site and stored in plastic bags with internal metal coatings for later analysis. Each composite sample was stored at 4 °C to avoid the degradation of organic matter.

Zooplankton was collected with a conical net (50 cm diameter; 333 µm mesh) equipped with a flowmeter to estimate the volume of filtered water. Zooplankton tows were done for 5 min at a depth of 60 cm. Once the biological material was recovered, it was preserved in glass bottles and fixed in a 4% solution of formaldehyde neutralized with sodium borate.

The speed of surface currents in the lagoon was determined with a drifting buoy launched during the sampling and later recovered about 20 min later; records of the start and end positions were done with a Garmin Astro 220 GPS (spatial resolution ± 5 m).

### Extraction of microplastics

#### Water

To detect microplastics in water, the sample (1 L) was passed through a pair of coupled sieves of 4.75 and 0.053 mm according to the method proposed by Rodrigues et al. (2018b). The retained particles were emptied into a beaker and subjected to organic matter oxidation with hydrogen peroxide (30% H_2_O_2_), at approximately 75 °C for 10 min, followed by density separation by adding a saturated zinc chloride solution (ZnCl_2_, 933 g/L), reagent considered one of the most effective for microplastics to float from aquatic samples, with high recovery rates (> 94%) (Konechnaya et al., 2020; Rodrigues et al., 2020). After 4 h, the supernatant was passed through a vacuum filtration system through a glass microfiber filter with a pore size of 1.6 µm. Subsequently, the filters were transferred to Petri dishes and dried in an oven at 40 °C for 1 week (Masura et al., 2015; Ng & Obbard, 2006; Rodrigues et al., 2018a). To avoid sample loss during the sieving, density separation, and filtration process, the sieves and walls of the containers used were carefully washed. Each filter was reviewed under a stereoscopic microscope to characterize (shape, color, and size) and quantify the retained plastic particles (Hidalgo-Ruz et al., 2012; Rochman et al., 2019). Microplastics were reported as the number of particles per liter of water (items/L).

#### Zooplankton

To detect microplastics in zooplankton, different organisms (e.g., copepods, chaetognaths, and luciferids) have been selected according to their feeding habits and trophic positions. The taxonomic identification of the zooplankton species was made using specialized literature (Boltovskoy, 1999a, 1999b; Castellani & Edwards, 2017; Johnson & Allen, 2012). For the extraction of microplastics, up to 1000 individuals of each type of organism were first manually separated, depending on their abundance (sampling stations with low numbers of animals were not analyzed) (Table 1). Afterward, the animals were placed on a 333-µm sieve and rinsed repeatedly with distilled water; later, the organisms were examined under a microscope to remove microplastics attached to the body. Then, they were placed into 20-mL vials for tissue digestion as proposed by Desforges et al. (2015), who after a series of experiments determined that nitric acid was the only solution that was able to finally digest the zooplankton tissue; nitric acid (100%, 5 mL) was added to each vial so that the organisms were completely covered, and then the vial was capped and heated in a water bath to ~ 80 °C for 65 to 120 min until the tissue was fully digested. Digestion was visually evaluated every 30 min until no biological materials were observed. The samples were then filtered through a 1.6-µm glass microfiber membrane. To avoid sample loss during the transition from digestion to filtration, the walls of vials were carefully washed. Once filtration was completed, the filters were dried under the same conditions described for water. The retained plastic particles were observed under stereoscopic and optical microscopes.

**Table 1:** Number of organisms used for the analysis of microplastics at each sampling station

Sampling station	Copepods	Chaetognaths	Luciferids
1	1000	NA	NA
2	56	NA	40
3	1000	56	100
4	253	NA	NA
5	1000	54	NA
6	1000	100	45
7	1000	92	42
8	1000	100	100
9	1000	100	100
10	1000	100	100
11	1000	100	100

*NA* not processed because the low number of animals

Additionally, microplastics were measured using a digital microscope and the HiView software. The concentration of microplastics in animals was expressed as the number of particles per individual (items/ind).

#### Sediments

The sediment samples were divided into two parts to analyze the microplastics and organic matter content. Microplastic analysis was performed according to the method proposed by Rodrigues et al. (2018a), which consists of homogenizing the sample with a spatula to obtain 500 g of wet weight. Samples were taken and dried at 40 °C for 5 days to determine the sediment dry weight, followed by a disintegration process with 400 mL of sodium hexametaphosphate (5.5 g/L) for 24 h and orbital shaking (80 rpm × 1 h). Each sediment sample was passed through two sieves (4.75 and 0.053 mm); the retained material was subjected to a density separation with ZnCl_2_ for 4 h. The subsequent procedure was the same as used in the water treatment (vacuum filtration, microscope analysis). To avoid sample loss during the procedure, the sieves and/or walls of the containers were carefully washed.

To assess any relationship with the abundance of microplastics, the organic matter content of the sediment was determined by oxidation with potassium dichromate (K_2_Cr_2_O_7_) and concentrated sulfuric acid (H_2_SO_4_), followed by titration with ferrous ammonium sulfate hexahydrate (Fe(NH_4_)_2_(SO_4_)_2_·6H_2_O) (0.5 N) (Gaudette & Flight, 1974). Since the microplastics and the organic matter have similar densities (Andrady, 2011; Avnimelech et al., 2001; Hoellein et al., 2019), one would expect they follow the same deposition pattern.

### FTIR analysis

The chemical characterization of all particles (100%) was done using a Fourier transform infrared with attenuated total reflectance spectrometer, with a microscope equipped with a liquid nitrogen-cooled mercury cadmium telluride (MCT) detector and a motorized stage that rapidly positions the sample at the correct position (µFTIR-ATR, Thermo Scientific™ Nicolet™ iN^TM^10). The particles were placed on a low-e glass slide holder plate with built-in locations, where each particle was compressed with a germanium crystal provided with a micro tip (350 µm), under the following conditions: reflectance mode with a resolution of wave number intervals of 675 and 4000 cm^−1^, within 4 cm^−1^, and 16 scans per particle with maximum spatial resolution, with a collection time of 3 s for all samples. Spectra were compared with two spectral libraries (Software OMNIC™ Picta™ and local plastic materials library), where only polymer matches greater than 70% similarity were accepted.

### Quality assurance/quality control (QA/QC)

Several quality control measures were implemented during the determination of microplastics to avoid exogenous contamination in the samples. For example, during their handling and analysis, laboratory coats and cotton clothing were used, as well as latex gloves. Plastic sampling instruments and laboratory equipment were avoided by opting for glass or metal items whenever possible. All solutions were filtered beforehand through a 1.6-µm glass microfiber membrane.

Before use, sample containers and glassware used in field and laboratory procedures were thoroughly washed and rinsed with filtered distilled water. The work area was cleaned and covered with aluminum foil before each analysis. Sample handling time was minimized, and secondary contamination from the air was avoided by covering the glass containers with aluminum foil. The filters were kept in glass Petri dishes during drying. For the microscopy analysis, an initial review was done with the Petri dishes closed, and only the lids were opened to handle the particles. To minimize airflow, laboratory doors and windows were kept closed and the exhaust fans were turned off. To determine the external contamination during the processing of the samples, a measurement was made in blanks of plastic particles. A blank was added for each batch of samples analyzed, following the same procedure as for the water, sediment, and zooplankton analyses. The contamination observed in the blanks was up to one particle, in which case it was characterized by color, shape, size, and polymer. The blank correction method consisted of subtracting the blank contribution as long as the color, shape, and polymer matched the associated samples (Kutralam-Muniasamy et al., 2023; Waddell et al., 2020).

### Data analysis

Owing to the nature of data, we used non-parametric tests to compare or relate data. The Spearman correlation coefficient was used to examine the correlation between two variables, i.e., the microplastic concentration in water and sediment; the Statistica software was used for this purpose. As well, the ANOSIM (similarity analysis) test was performed to determine if significant differences exist among the zooplankton groups analyzed and between water microplastic concentrations of the marine-influenced area and the estuarine zone. The software used for this purpose was the PRIMERv7 (Clarke & Gorley, 2015).

## Results and discussion

### Characteristics of microplastics: Shape, color, size, and polymer type

**Fig. 2:**
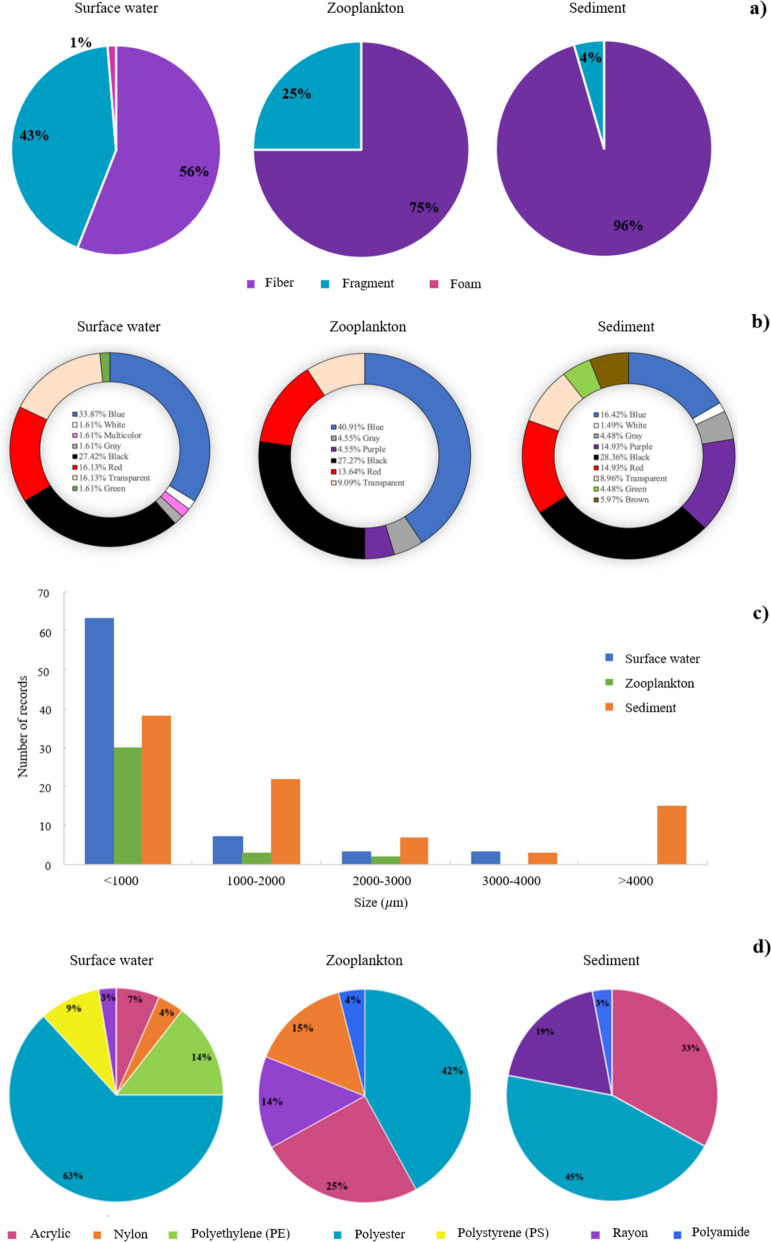
Characteristics of microplastics observed in surface waters, zooplankton, and sediments in the Sontecomapan lagoon according to **a** shape, **b** color, **c** size, and **d** type of polymer

Three types of microplastic shapes were observed in this study: fibers, fragments, and foams. In the water, fibers represented 56%, followed by fragments (42.7%), and foams with low abundance (1.3%). In zooplankton, only fibers (75%) and fragments (25%) were detected. In sediments, fibers represented a high percentage (95.6%), followed by fragments (4.4%) (Fig. 2a and Supplementary Material Table S1). The dominance of fibers in the three compartments could have originated from synthetic fabrics (clothing or carpets), fishing nets, fishing rods, and ropes. The fragments came from the breakdown of jars or beverage bottles, whereas the foams derive from the rupture of foam cups and food packaging (Acharya et al., 2021; Hossain et al., 2022).

The predominant color of microplastics in surface waters was blue (33.87%), followed by black (27.42%), as in the zooplankton (40.91% and 27.27%, respectively) which suggests a close relationship between the particles present in the environment and those that the organisms ingest. Nine colors of microplastics were found in the sediment samples (blue, white, gray, purple, black, red, transparent, green, and brown); the dominant color was black (28.36%), followed by blue (16.42%) (Fig. 2b and Supplementary Material Table S1). Previous studies have also shown that blue is the predominant color of particles in water (Abayomi et al., 2017; Sawalman et al., 2021; Zheng et al., 2019), whereas in the sediment compartment, the black is dominant (Gray et al., 2018; Nematollahi et al., 2020; Zheng et al., 2019).

The size of microplastics in the water samples varied between 20 and 4730 µm, and 92.1% of the particles were in the first two size classes (< 1000 µm and 1000–2000 µm) (Fig. 2c and Supplementary Material Table S1). In accordance, previous studies in China and Bangladesh found a high proportion of small-sized microplastics in water (Hossain et al., 2022; Jiang et al., 2019). In the zooplankton, microplastic size varied between 10 and 2600 µm, the average value was 450 ± 630 µm, and the smallest microplastics (< 1000 µm) were the most common. This same pattern was observed in China and Malaysia, where zooplankton organisms ingested small-sized microplastics (Sun et al., 2018a, 2018b; Taha et al., 2021), because small particles are more likely to be ingested (Hastuti et al., 2019). The largest microplastics were found in the sediment, with an average size of 1610 ± 1366 µm and a size spectrum from 100 to 4250 µm. Larger and denser particles are more likely to be deposited in this compartment (Liu et al., 2022).

FTIR analysis showed that 72% of the analyzed particles were plastic polymers. The chemical composition of the polymers in surface water indicated the dominance of polyester (63%) and polyethylene (PE) (14%); polystyrene (PS), rayon, acrylic, and nylon were found at a percentage of less than 9% (Fig. 2d); in addition, the presence of poly(diallyl phthalate), a plasticizer whose discovery demonstrates plastic contamination, was recorded. The polymers detected in zooplankton organisms were polyester (42%), acrylic (25%), nylon (15%), and in a lower percentage, rayon and polyamide. The spectra of the particles in sediment samples indicated the presence of polyester (45%) and acrylic (33%), along with rayon (19%) and polyamide (3%) (Fig. 2d and Supplementary Material Table S1). The polymers found here are associated with the production of various objects: polyester is mainly used in the production of clothing, which can release between 4.6 × 10^10^ and 8.9 × 10^11^ particles per gram of textile during washing (Yang et al., 2024): PE in reusable bags, agricultural films, toys, and bottles; PS in food packaging, foam packaging, plastic tableware, disposable cups, plates, and cutlery; and acrylic and nylon in clothing, ropes, and nets (Acharya et al., 2021; Hernandez et al., 2017; Jones et al., 2020; Nolasco et al., 2022). The waste generation in the study area is associated with the economic activities within the lagoon and its surroundings, such as fishing, tourism, and agriculture. Plastics can arrive through continental tributaries from small towns surrounding the lagoon, such as Sontecomapan village, Coxcoapan, and La Palma (Fig. 1), or even from more distant sites such as Catemaco.

### Water

**Fig. 3:**
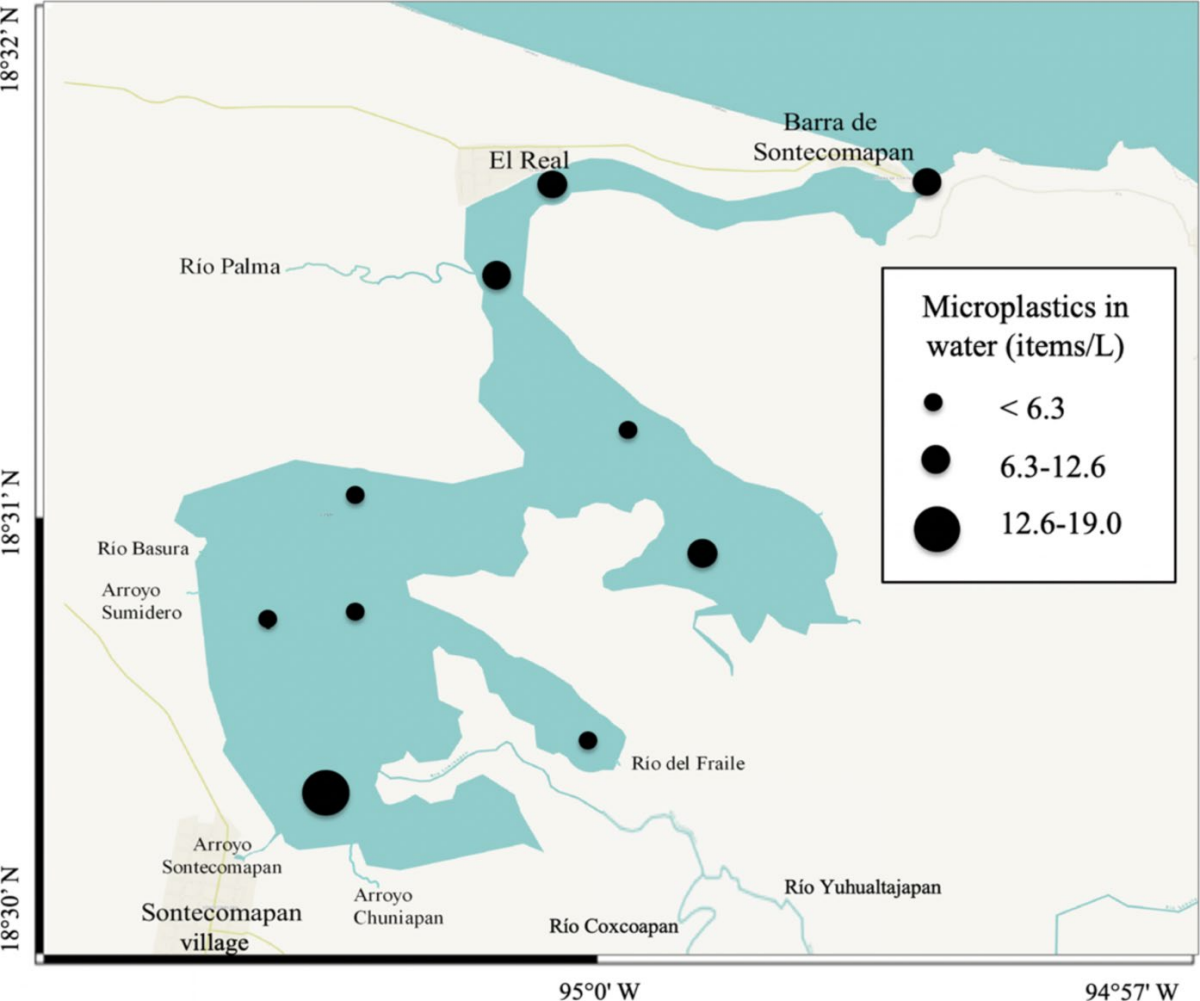
Concentration of microplastics in surface water of the Sontecomapan lagoon

Microplastics were found in all water samples (Fig. 3); the average concentration was 7.5 ± 5.3 items/L, with a maximum of 19 items/L (Supplementary Material Table S2). Regarding spatial distribution, one could expect differences between the marine-influenced area (> 25 psu) and the estuarine zone (< 25 psu); however, results of the ANOSIM test indicated no spatial differences between the estuarine zones (*R* =  − 0.133, *p* > 0.05) in the concentration of microplastics. Even in the case of significant differences, it is very difficult to establish the origin of microplastics in the lagoon because they can also be transported by the wind, the presence of some small rivers near the mouth, and the human settlements around the lagoon.

In other protected coastal systems, the recorded values have been lower than those found here (Table 2), with a difference of up to four orders of magnitude. For example, in the Río Lagartos coastal lagoon, Yucatán (Mexico), concentrations of 3 to 4 (× 10^−4^) items/L were found (Quesadas-Rojas et al., 2021). On the Caribbean coast of Colombia, in the Ciénaga Grande de Santa Marta lagoon complex, concentrations of 0.0 to 0.3 items/L were found (Garcés-Ordóñez et al., 2022a). However, in those cases, the authors sieved the water samples through a 200- or 300-µm mesh (Table 2), which could be the chief cause of the differences to the present study. In estuarine sites without any protection category and surrounded by agricultural-industrial or highly populated areas, concentrations of microplastics have been found in the range of 0.00 to 0.17 items/L (Haddout et al., 2022; Liu et al., 2022). Again, in those studies, the water samples were sieved through a 300-µm mesh, and salts were added at a lower density (CaCl_2_, 1.34 g/cm^3^; NaCl, 2.16 g/cm^3^) than that used here (ZnCl_2_, 2.91 g/cm^3^).

**Table 2:** Microplastic concentration in water in several ecosystems around the world

References	Sampling method and laboratory analysis	Microplastics (items/L)	Country	Ecosystem (protection category)
This study	Composite sample with a bucket (10 L)	average 7.5	Mexico	Coastal lagoon (Ramsar site/Protected Natural Area)
	Sieving (53 µm mesh size)			
	Organic matter oxidation (H_2_O_2_)			
	Density separation (ZnCl_2_)			
	Vacuum filtration system			
Quesadas-Rojas et al. (2021)	Zooplankton net (200 µm mesh size)	0.0003 − 0.0004	Mexico	Coastal lagoon (Biosphere Reserve)
Sánchez-Hernández et al. (2021)	Glass bottles (5 L)	111	Mexico	Tecolutla Estuary (without any protection category)
	Visual extraction of microplastics < 5 mm			
	Vacuum filtered			
Garcés-Ordóñez et al. (2022a)	Metal bucket (100 L) Sieving (300 µm mesh size) Visually inspected under a stereomicroscope	0.0–0.3	Colombia	Lagoon complex (RAMSAR site/Biosphere Reserve)
Haddout et al. (2022)	Steel sampler (20 L) Sieving (300 µm mesh size)	0.0–0.17	Morocco	Estuary (without any protection category)
	Organic matter oxidation (H_2_O_2_)			
	Vacuum filtration system			
Liu et al. (2022)	Metal bucket (100 L)	0.02	Netherlands	Estuary (without any protection category)
	Sieving (5600, 425, 75, and 40 µm mesh size)			
	Organic matter oxidation (H_2_O_2_)			
	Density separation (CaCl_2_)			
	Vacuum filtration system			
Ragoobur et al. (2023)	Glass bottle (2 L) Organic matter oxidation (H_2_O_2_) Vacuum filtration system	7–1290	Mauritius island	Mauritius Estuary (without any protection category

In other estuaries of Mexico and the Mauritius islands, concentrations of microplastics of the order of 10^2^ to 10^3^ items/L were found (Ragoobur et al., 2023; Sánchez-Hernández et al., 2021), higher than that recorded here. Those studies used field and laboratory methods similar to those employed here, which makes the comparison between sites more reliable. Therefore, the higher concentration of microplastics in those estuaries may be due to the anthropic impact of the higher population density of the towns nearby (between 12 and 25 thousand inhabitants) (INEGI, 2021; Statistics Mauritius, 2019).

A study joining microplastic data from marine and coastal waters, marine protected areas, and buffer zones at a global level classified the values into four quartiles: Q1: 0.000042–0.2; Q2: 0.2–1.7; Q3: 1.7–144.7; and Q4: 144.7–809,000 items/m^3^ (Nunes et al., 2023a); the mean value found in this study (7500 items/m^3^) place the Sontecomapan estuary within the Q4 quartile, suggesting a high level of contamination. Again, the methods employed in the works are different.

In addition to the methodological differences, the high microplastic concentration detected in the water could result from the resuspension of particles initially deposited in the sediments. Wind and waves can resuspend microplastics into the water column, a common process in shallow aquatic systems (Brand et al., 2015; Vermeiren et al., 2016).

### Zooplankton

Zooplankters are highly sensitive to contamination by microplastic; their daily migrations in the water column make them vectors of particle contamination in aquatic environments (Wright et al., 2013). In the study area, the mean (± *SD*) microplastic contamination and the standard error (*SE*) were as follows: 0.002 ± 0.005 items/ind, *SE* = 0.000006 for copepods; 0.011 ± 0.011 items/ind, *SE* = 0.0004 for chaetognaths; and 0.019 ± 0.016 items/ind, *SE* = 0.001 for luciferids (Supplementary Material Table S2). The mean values were significantly lower in the copepods than in the other two other groups, according to the ANOSIM test (copepods vs chaetognaths *R* = 0.73; copepods vs luciferids *R* = 0.63; chaetognaths vs luciferids *R* = 0.60, all with *p* < 0.05).

**Fig. 4:**
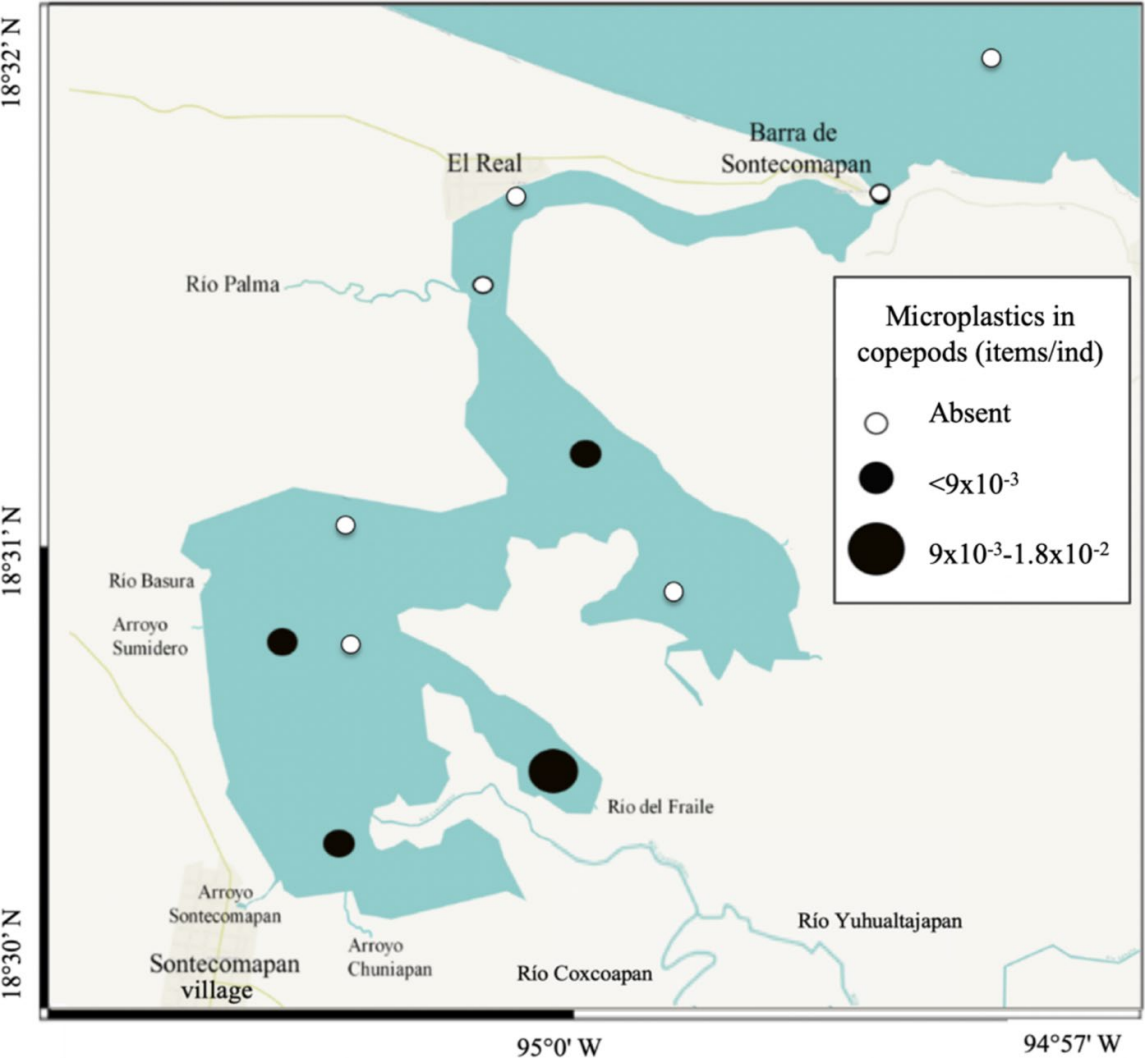
Distribution of microplastics ingested by copepods in the Sontecomapan lagoon

**Fig. 5:**
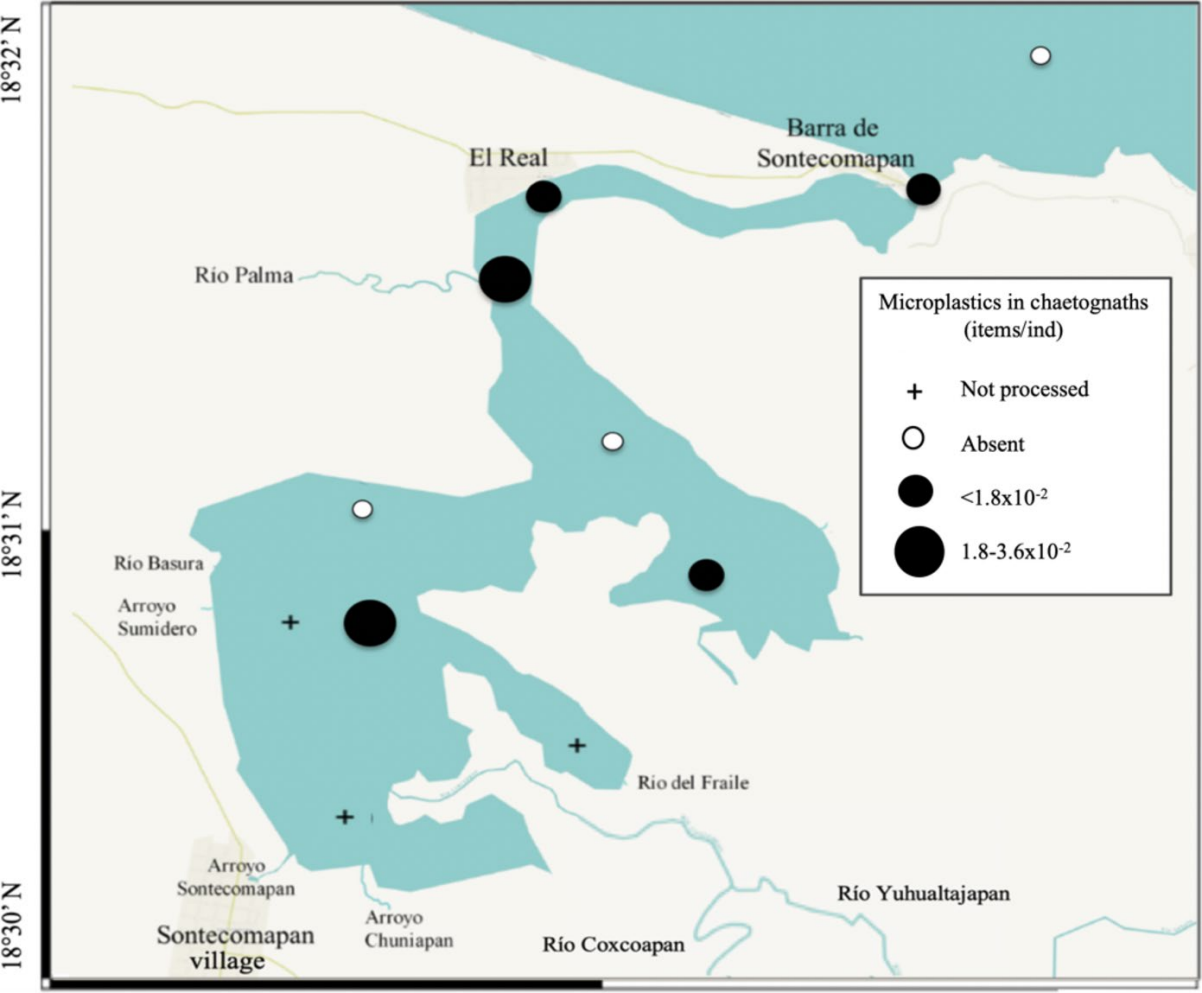
Distribution of microplastics ingested by chaetognaths in the Sontecomapan lagoon

**Fig. 6:**
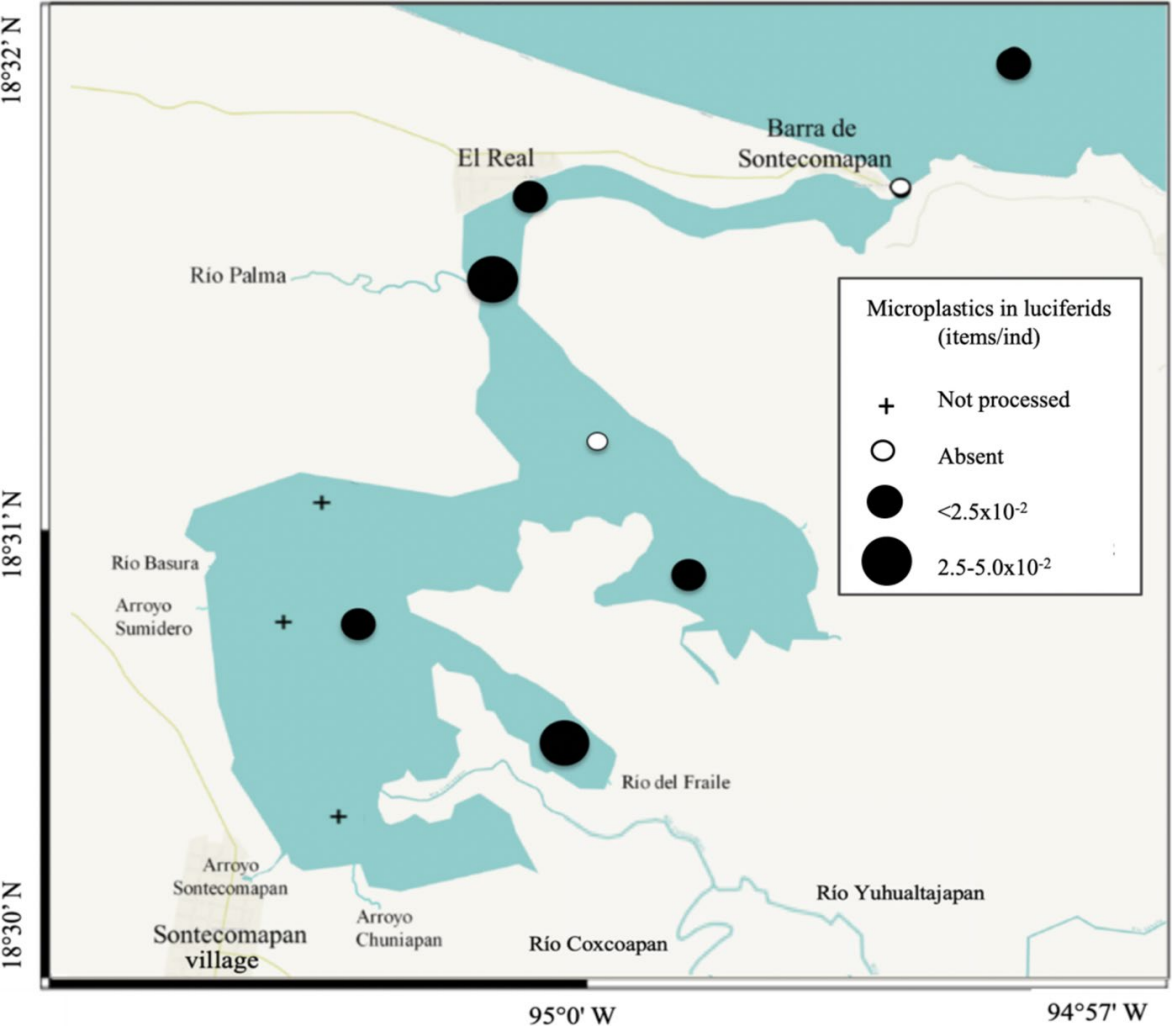
Distribution of microplastics ingested by luciferids in the Sontecomapan lagoon

The variability in the number of particles consumed by the three groups in the order of 10^−3^ to 10^−2^ items/ind (Figs. 4, 5, and 6) may result from the differences in their feeding habits, trophic position, and function in the ecosystem. Copepods, represented mainly by the species *Acartia tonsa* in the study area, are considered suspension filter feeders since they consume phytoplankton through the creation of feeding currents, but they also can actively catch small prey; chaetognaths are primarily carnivorous predators, consuming mainly copepods; and luciferids are omnivorous and feed on small crustaceans and microalgae (Cházaro-Olvera et al., 2017; Saiz & Kiørboe, 1995; Sanvicente-Añorve et al., 2020; Terazaki, 2000). We propose that the higher concentration of microplastics in chaetognaths and luciferids is because they could acquire microplastics both directly and indirectly, that is, through a direct capture of the microplastic particle due to confusion or the ingestion of previously contaminated prey (Sun et al., 2017).

Previous studies have documented the potential of these zooplankters to ingest microplastics in their natural environment (Amin et al., 2020; Goswami et al., 2023; Kosore et al., 2018; Zavala-Alarcón et al., 2023). Zavala-Alarcón et al. (2023) found microplastic concentrations of 10^−2^ items/ind in copepods for the central Mexican Pacific, and the authenticity of the particles was verified by Raman spectroscopy. Using different methods, Montoya-Melgoza et al (2024) showed concentrations of 10^−1^ items/ind in copepods in the southern Gulf of Mexico.

However, only in the Yellow Sea and East China Sea have the three groups been analyzed simultaneously; there, microplastic concentration per individual (copepods < chaetognaths < luciferids) was at slightly higher orders of magnitude (10^−2^ to 10^−1^) (Sun et al., 2018a, 2018b) than in this study; this may reflect greater anthropogenic pressure from neighboring large cities, such as Shanghai, Hangzhou, and Qingdao.

In general, studies regarding the contamination of zooplankton by microplastics in estuaries are very scarce. In the Terengganu estuary, east Malaysia, an average concentration of ~ 10^−1^ items/ind was recorded (Taha et al., 2021), higher than that recorded in this study (Table 3). These differences may reflect the large river discharge formed by the estuary itself and the economic activity associated with the city of Kuala Terengganu with 286,317 inhabitants (Bagheri et al., 2023).

**Table 3:** Microplastic concentration in zooplankton in several ecosystems around the world

References	Sampling method and laboratory analysis	Microplastics (items/ind)	Country	Ecosystem (protection category)
This study	Bongo nets (333 µm mesh size)	Chaetognaths 0.019	Mexico	Coastal lagoon (Ramsar site/Protected Natural Area)
	Remove microplastics attached to the body	Luciferids 0.011 Copepods 0.002		
	Tissue digestion (HNO_3_)			
	Water bath (80 °C × 65–120 min)			
	Vacuum filtration system			
	Visually inspected under a stereomicroscope			
Sun et al. (2018a)	Bongo nets (500 µm mesh size)	Chaetognaths ~ 0.09	China	Yellow Sea (without any protection category)
	Individuals were rinsed with deionized water	Luciferids ~ 0.09 Copepods ~ 0.08		
	Tissue digestion (HNO_3_)			
	Water bath (80 °C × 3 h)			
	Vacuum filtration system Visually inspected under a stereomicroscope			
Sun et al. (2018b)	Bongo nets (500 µm mesh size)	Chaetognaths ~ 0.2	China	The East China Sea (without any protection category)
	Individuals were rinsed with deionized water	Luciferids ~ 0.3 Copepods 0.13		
	Tissue digestion (HNO_3_)			
	Water bath (80 °C × 3 h)			
	Vacuum filtration system Visually inspected under a stereomicroscope			
Taha et al. (2021)	Remove microplastics attached to the body	Chaetognaths 0.20 Copepods < 0.05	Malaysia	Terengganu estuary and offshore waters (without any protection category)
	Tissue digestion (HNO_3_)			
	Water bath (80 °C × 30 min)			
	Visually inspected under a stereomicroscope			

**Fig. 7:**
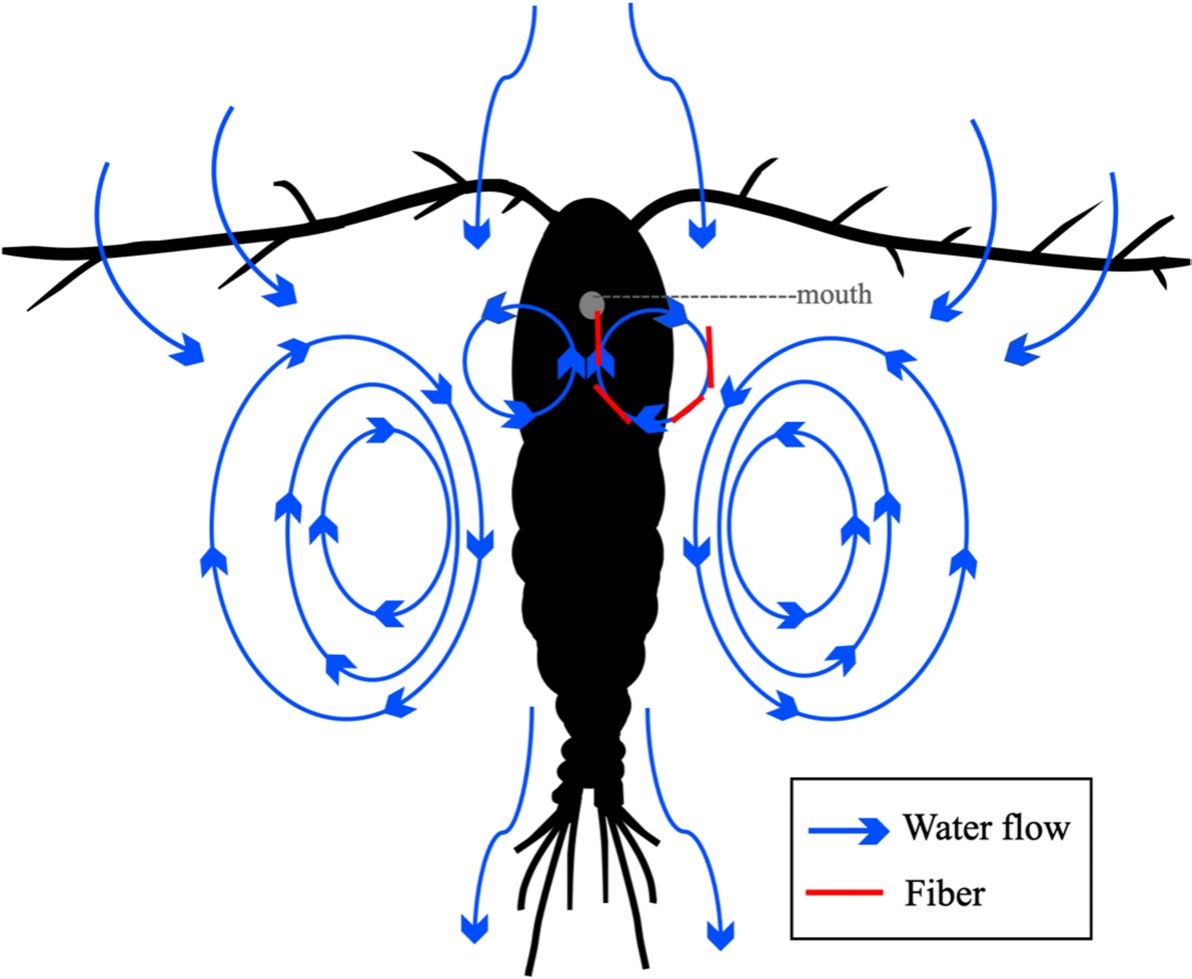
Hypothetical scheme of fiber ingestion by *Acartia tonsa*

In the present study, copepods consumed only fibers, whereas chaetognaths and luciferids ingested fibers and fragments. In general, this difference in the shape of particles in these animals may reflect the size of their natural prey, the time spent in the digestive system, or the occurrence of a dominant form in the environment they inhabit (Botterell et al., 2020). Consistent with this study, Botterell et al. (2020) found only fibers in the copepod *A. tonsa*; they speculated that the species may confuse the fibers with chain-forming diatoms. We think that when the species feeds by filtering, the fibers are transported tangentially to the lines of the microcurrents generated by the copepod in such a way that they arrive almost perpendicularly to the mouth and penetrate the digestive tract directly (Fig. 7). The diameter (10 µm) of the ingested fibers was within the range of the distance between setules on the maxilla (3 to 15 µm) recorded in *A. clausi* (Nival & Nival, 1976). This could explain why *A. tonsa* consumed only fibers.

The relationship analysis between microplastic concentration in water and zooplankton (including the three groups) indicated a non-significant association (Spearman test, *r* = 0.13, *n* = 10, *p* = 0.70). Two causes may account for this result: the first concerns the bioavailability of microplastics in water, this is, the proportion potentially available for an organism, which depends on the characteristics of the microplastics (shape, size, color, abundance) and the feeding mode of the animals (Botterell et al., 2019, 2020; Wright et al., 2013); and the second reason involves the level of water turbulence and the ability of each type of organism to trap food; turbulence may cause confusion between microplastics and motile prey resulting in accidental ingestion of microplastics (Fuchs & Gerbi, 2016; Sanvicente-Añorve et al., 2023).

### Sediments

The average concentration of microplastics in the study area was 8.5 items/kg, with a maximum value of 43.0 items/kg (Supplementary Material Table S2). These values are comparable with the records of other Ramsar wetlands worldwide. Thus, in a mangrove ecosystem on the north coast of the Persian Gulf, values of 19.5 to 34.5 items/kg were recorded (Naji et al., 2019); in the Ria Formosa lagoon in southern Portugal, the average was 34 items/kg (Cozzolino et al., 2020), and in the Ciénaga Grande de Santa Marta lagoon complex in Colombia, the average was 2.4 × 10^−1^ items/kg (Garcés-Ordóñez et al., 2022a). In contrast, in the Bahía Blanca estuary in Argentina, a highly anthropized site, the average concentration was 1693 items/kg (Arias et al., 2023) (Table 4). Hence, the concentration of microplastics in the protected sites was in the order of 10^−1^ to 10^1^, whereas in the high-impact places, they were in the order of 10^3^. The proximity to populated areas, wastewater discharges, industrial establishments, and the degree of exposure of the coast to waves are among the leading causes of high levels of microplastics in sediments (Blumenröder et al., 2017).

**Table 4:** Microplastic concentration in sediments in several ecosystems around the world

References	Sampling method and laboratory analysis	Microplastics (items/kg)	Country	Ecosystem (protection category)
This study	Van Veen grab	Average 8.5	Mexico	Coastal lagoon (Ramsar site/Protected Natural Area)
	Disintegration (sodium hexametaphosphate)			
	Sieving (4.75 and 0.053 mm mesh size)			
	Density separation (ZnCl_2_)			
	Vacuum filtration Visually inspected under a stereomicroscope			
Naji et al. (2019)	Stainless-steel shovel (Top 5 cm)	19.5–34.5	Iran	Mangrove ecosystem (The Hara Protected Area)
	Sieving (1 mm mesh size)			
	Density separation (NaCl and NaI) Vacuum filtration Visually inspected under a stereomicroscope			
Cozzolino et al. (2020)	Plastic box corer (2–3 cm of sediment surface)	Average 34	Portugal	Ria Formosa lagoon (Protection Area/Natural Park/Ramsar site)
	Density separation (NaCl) Vacuum filtration Visually inspected under a stereomicroscope			
Garcés-Ordóñez et al. (2022a)	Stainless steel Van Veen dredge	Average 0.24	Colombia	Lagoon complex (Ramsar site/Biosphere Reserve)
	Sieving (5 mm and 0.5 mm mesh size)			
	Examined under the stereomicroscope			
Arias et al. (2023)	Metallic sampling cores (10 cm depth)	Average 1693	Argentina	Bahía Blanca estuary (without any protection category)
	Sieving (1–5 mm, 5660 y 1000 µm mesh size)			
	Examined under the stereomicroscope			

A recent review including sediment microplastic data of many protected and non-protected areas classified the concentration values into four quartiles: Q1: 0.1–22; Q2: 22–106.7; Q3: 106.7–362.1; Q4: 362.1–145,435 items/kg (Nunes et al., 2023b). The average value found in this study (8.5 items/kg) is placed at the Q1, within the low levels of contamination.

**Fig. 8:**
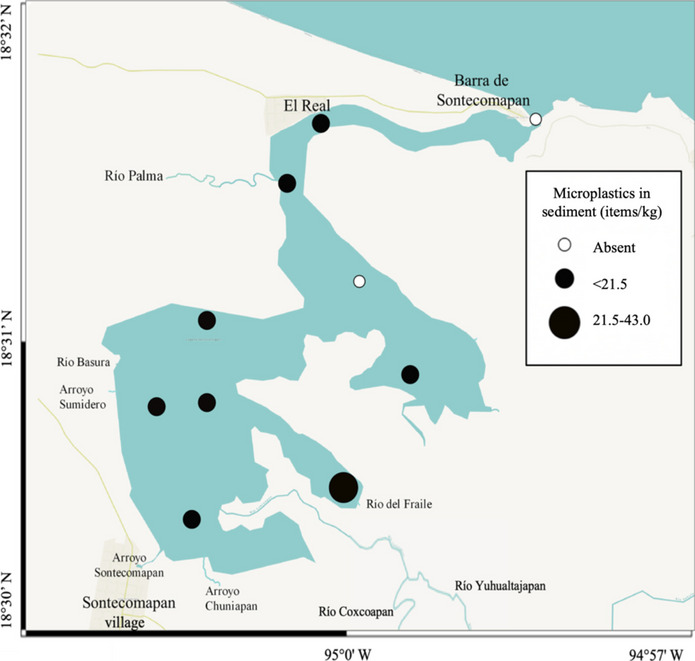
Concentration of microplastics in the sediments of the Sontecomapan lagoon

**Fig. 9:**
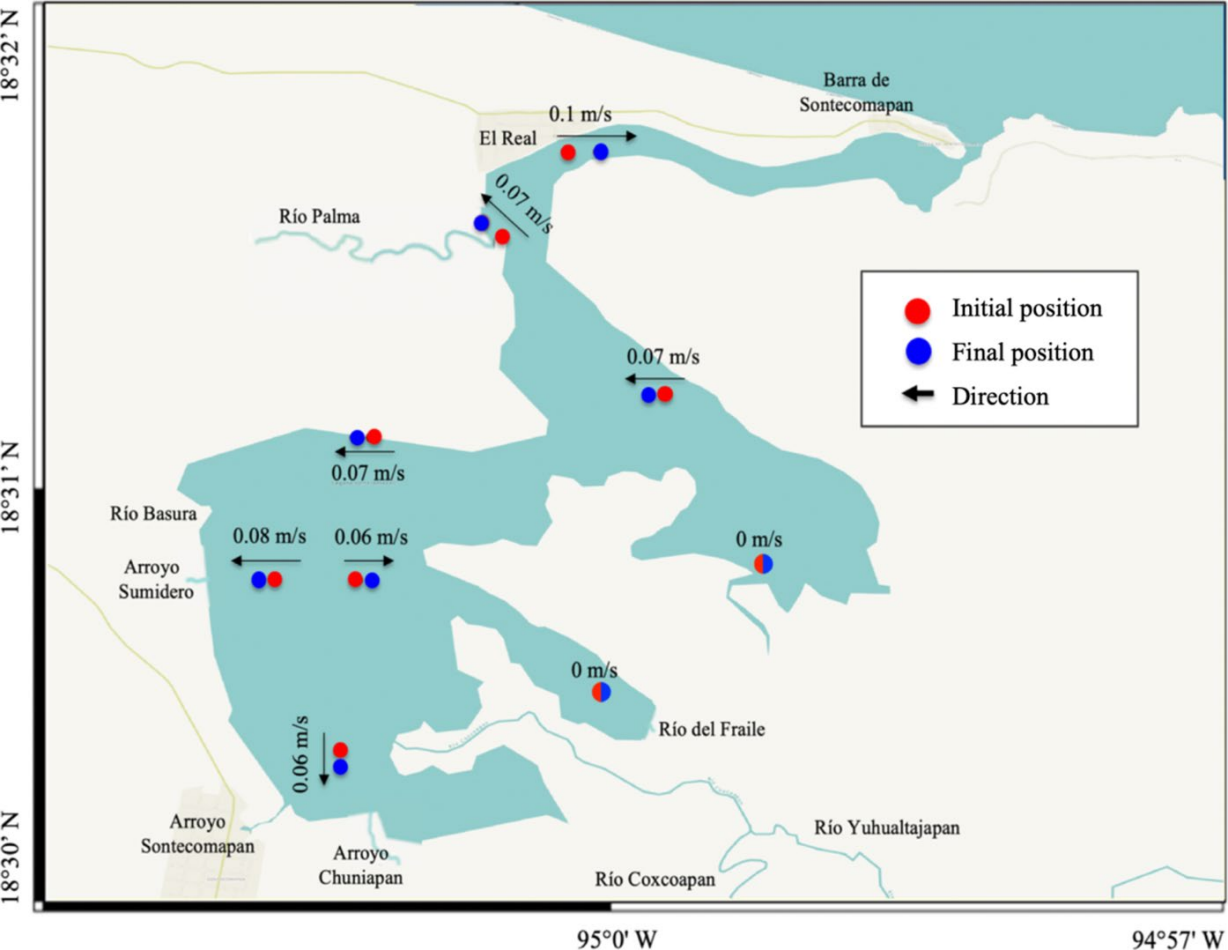
Direction and speed of drifting buoys in the Sontecomapan lagoon

In this study, the site of the highest accumulation of microplastics in the sedimentary compartment (43.0 items/kg) was in a nook in the southeastern section of the lagoon (station 2, Fig. 1), where no current was detected (Figs. 8 and 9); this suggests that areas with zero or low current velocity are more prone to accumulate microplastics and therefore represent a greater risk for benthic organisms. Indeed, water and sediment concentrations of microplastics did not show a significant correlation (Spearman test, *r* =  − 0.30,* n* = 10, *p* = 0.38) because of the influence of water currents.

The positive association (Spearman test, *r* = 0.67, *n* = 10, *p* = 0.03) found in this study between the organic matter content and the concentration of microplastics in the sediments may result from the similarity in density between the two elements. The average density of organic matter is 1.25 g/cm^3^ (Avnimelech et al., 2001), similar to that of polystyrene fragments (1.05 g/cm^3^) and synthetic fibers (1.17 g/cm^3^) (Andrady, 2011; Hoellein et al., 2019). The two types of particles have similar transport and deposition characteristics because they are subjected to the same hydrodynamic forces that induce the sinking or resuspension of particles (Hoellein et al., 2019; Vincent & Hoellein, 2021). The density of particles is the main factor in deposition dynamics, but other features that alter the deposition rates of microplastics are the size, shape, or colonization of microbial biofilms (Hoellein et al., 2019). Furthermore, since microplastics are hardly degraded, they can reach longer distances than particulate organic matter (Vincent & Hoellein, 2021).

The Mexican Government has encouraged its inhabitants to follow certain measures to reduce the usage of plastics, such as minimizing the use of disposable products and choosing returnable packaging, preferring products whose plastic packaging has the recyclable or recycled logo, reuse supermarket bags for shopping, or buy cleaners and cleaning products whose label indicates that they are biodegradable. The creation of MPA is a good measure that is providing favorable results to protect ecosystems; however, there is still much to do in terms of ecological awareness. The government, companies, educational centers, and individuals have to work together to maintain a healthy environment, which also means human well-being.

### Trend of microplastics in the Sontecomapan lagoon

In the Sontecomapan lagoon, the entry of microplastics or larger plastics can occur through three routes: incorporation of continental water by discharge from rivers and streams that surround the system, atmospheric transport due to the capacity of the wind to transport microparticles from surrounding land areas (Zhang et al., 2020), and entry of seawater through tidal currents. The difference between this and other estuarine systems is the amount of microplastics arriving into the system, which largely depends on the socioeconomic activities of each region and population density. Among the economic activities that promote the generation of plastic waste in the area are fishing, in which the nylon nets and traps employed release fibers; crop production, in which a wide variety of plastic materials are used (greenhouses, mulch, irrigation systems and planters); and tourism activity with its infrastructure and users, added to the presence of small surrounding towns, whose waste in some indeterminate quantity reach the rivers and streams that connect with the lagoon as well as directly to this ecosystem.

**Fig. 10:**
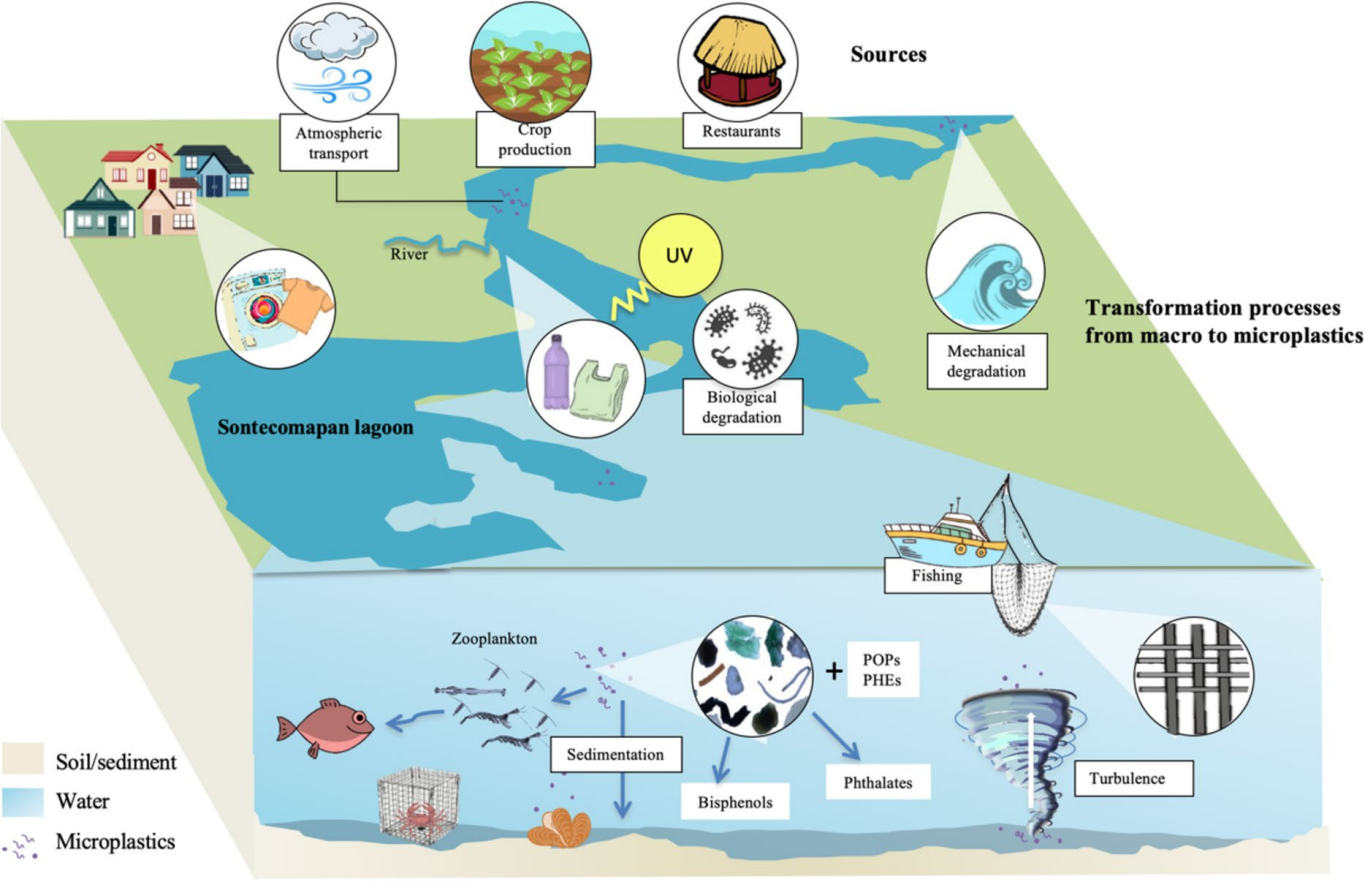
Hypothetical scheme of sources, processes, transport, and fate of microplastics in the Sontecomapan lagoon

Likewise, microplastics can enter the lagoon as synthetic fibers from clothing or larger plastics that can be transformed into microplastics by photodegradation (UV radiation), mechanical force (wind, waves, and tides), or biological degradation (Andrady, 2011). Once inside the lagoon, microplastics can remain floating on the water, being bioavailable to pelagic organisms such as zooplankton, which will allow this contaminant to enter the ecosystem’s food web or continue its trajectory until be deposited in the sediments (Fig. 10). Once in the sediment, microplastics can remain stored or be ingested by benthic organisms along with or instead of their food.

In general, a clear difference could be observed in the occurrence of plastic particles in the water and sediment, as a response to the residence time of the microplastics in each compartment. Surface waters reflect both constant and transient inputs of these particles, which will eventually be colonized by organisms or aggregates of organic waste. This colonization will increase their apparent density, causing them to sink in the water column (Lobelle and Cunliffe, 2011; Abidli et al., 2018). Once microplastics enter the sedimentary compartment, they can be resuspended by processes such as turbulence, freshwater inflows, or storms that create vertical mixing; a decrease in its adsorbed material can cause floating–sinking cycles (Andrady, 2017; Cole et al., 2011; Ye & Andrady, 1991). However, sediments represent the accumulation process of this group of contaminants over time, which is why they are considered a long-term sink (Van Cauwenberghe et al., 2015). The permanence of microplastics in the system could allow the release of additives from the particles (such as bisphenols and phthalates) or the adsorption of persistent organic pollutants (POPs) and potentially hazardous elements (PHEs) found in the medium (Anderson et al., 2016). Combining all these elements allows us to propose a model of the primary sources, processes, and destinations that affect microplastics in the Sontecomapan lagoon (Fig. 10).

According to the microplastic concentrations found in the study area, our hypothesis concerning the three compartments was rejected, since the following trend is denoted considering the abundance of particles in the system: water > sediment > zooplankton, so it is of utmost importance that the water compartment is under surveillance. In general terms, the sediments and zooplankton biota of this lagoon ecosystem are less contaminated by microplastics in contrast to the water column, which can be considered to have a high level of contamination by microplastics, so our hypothesis about the low degree of contamination would only be fulfilled in sediments and biota. However, the results obtained in this study should be taken with caution, as with other works (Quesadas-Rojas et al., 2021; Garcés-Ordóñez et al., 2022a; Table 4), given the evolution of the methods and the technological advances that exist worldwide to achieve international methodological standardization and validation.

## Conclusions

This study assessed the degree of contamination of microplastics in the abiotic (surface water and sediment) and biotic (zooplankters) compartments of the coastal lagoon of Sontecomapan, a Ramsar site facing the southern Gulf of Mexico.

In general, concentrations of microplastics in the sediment were low (< 43 items/kg). This is comparable to other Ramsar wetlands worldwide, but up to two orders of magnitude lower than sites with high anthropic impact. In surface water, microplastics had higher values than those registered in other protected coastal systems in the world probably due to differences in the methods employed, that is, the size of the mesh pore and the use of salts of different densities. We reinforce the necessity to standardize the methods used to detect and quantify this pollutant to make reliable comparisons among sites.

In the biotic compartment, the ingestion of microplastics is confirmed in wild populations of the three zooplankton groups analyzed: copepods, luciferids, and chaetognaths. These animals differ in their feeding habits and food intake, thus suggesting that plastic particles are in several levels of the pelagic trophic web. The values here recorded in zooplankton were lower than in other places worldwide more prone to the influence of human activities.

The analysis of sources of origin, degradation processes, and transport of plastic particles allowed the formulation of a hypothetical scheme of microplastics’ origin and environmental fate in the Sontecomapan lagoon, which could provide graphic information for future studies.

The results of this research provide a basic understanding of the degree of contamination by microplastics in the Sontecomapan lagoon and provide relevant information to the competent authorities to establish appropriate measurements to regulate this contaminant.

## Supplementary information

Below is the link to the electronic supplementary material.

Supplementary file1 (DOCX 24 KB)

## Data Availability

Data are available in the Supplementary Information.
