# Supplementary material for: Microplastic contamination in three environmental compartments of a coastal lagoon in the southern Gulf of Mexico
Source: Environ Monit Assess. 2024 Oct 4;196(11):1012. doi: 10.1007/s10661-024-13156-2 (PMC11452496; doi:10.1007/s10661-024-13156-2)
Supplement: Supplementary file 1 — Supplementary file1 (DOCX 24 KB) [file 10661_2024_13156_MOESM1_ESM.docx]

**Table S1.** Percentage of microplastics recorded at each sampling site in the Sontecompan lagoon according to different characteristics.

| SHAPES | | | | | | | | | | | | | |
| --- | --- | --- | --- | --- | --- | --- | --- | --- | --- | --- | --- | --- | --- |
| Station | | 1 | 2 | 3 | 4 | 5 | 6 | 7 | 8 | 9 | 10 | 11 |  |
| Water | Fiber | 50.0 | 25.0 | 55.2 | 28.6 | 62.5 | 50.0 | 57.1 | 77.8 | 76.9 | 76.9 | NA |  |
|  | Fragment | 50.0 | 75.0 | 32 | 71.4 | 37.5 | 50.0 | 42.9 | 22.2 | 23.1 | 23.1 | NA |  |
|  | Foam | 0.0 | 0.0 | 12.8 | 0.0 | 0.0 | 0.0 | 0.0 | 0.0 | 0.0 | 0.0 | NA |  |
| Zooplankton | Fiber | 100.0 | 100.0 | 42.9 | 100.0 | 0.0 | 72.0 | 100.0 | 55.0 | 80.0 | 0.0 | 100.0 |  |
|  | Fragment | 0.0 | 0.0 | 57.1 | 0.0 | 0.0 | 28.0 | 0.0 | 45.0 | 20.0 | 100.0 | 0.0 |  |
| Sediment | Fiber | 100.0 | 65.0 | 100.0 | 100.0 | 100.0 | 100.0 | 0.0 | 100.0 | 100.0 | 0.0 | NA |  |
|  | Fragment | 0.0 | 35.0 | 0.0 | 0.0 | 0.0 | 0.0 | 0.0 | 0.0 | 0.0 | 0.0 | NA |  |
| COLORS | | | | | | | | | | | | | |
| Water | Blue | 14.0 | 5.0 | 28.3 | 67.8 | 60.0 | 54.1 | 27.1 | 36.9 | 26.4 | 19.1 | NA |  |
|  | White | 0.0 | 0.0 | 9.5 | 0.0 | 0.0 | 6.6 | 0.0 | 0.0 | 0.0 | 0.0 | NA |  |
|  | Multicolor | 4.6 | 0.0 | 0.0 | 0.0 | 0.0 | 0.0 | 5.6 | 0.0 | 5.9 | 0.0 | NA |  |
|  | Gray | 0.0 | 0.0 | 0.0 | 16.1 | 0.0 | 0.0 | 0.0 | 0.0 | 0.0 | 0.0 | NA |  |
|  | Black | 25.6 | 30.0 | 29.2 | 0.0 | 40.0 | 23.1 | 0.0 | 25.0 | 50.7 | 50.7 | NA |  |
|  | Red | 29.9 | 35.0 | 33.0 | 0.0 | 0.0 | 16.2 | 0.0 | 0.0 | 17.0 | 30.2 | NA |  |
|  | Transparent | 25.9 | 30.0 | 0.0 | 0.0 | 0.0 | 0.0 | 67.3 | 38.1 | 0.0 | 0.0 | NA |  |
|  | Green | 0.0 | 0.0 | 0.0 | 16.1 | 0.0 | 0.0 | 0.0 | 0.0 | 0.0 | 0.0 | NA |  |
| Zooplankton | Blue | 33.3 | 79.0 | 16.5 | 62.0 | 0.0 | 50.0 | 66.7 | 25.6 | 35.1 | 0.0 | 0.0 |  |
|  | Gray | 0.0 | 0.0 | 0.0 | 10.0 | 0.0 | 0.0 | 0.0 | 0.0 | 30.8 | 0.0 | 0.0 |  |
|  | Purple | 0.0 | 21.0 | 0.0 | 0.0 | 0.0 | 0.0 | 0.0 | 20.0 | 0.0 | 0.0 | 0.0 |  |
|  | Black | 0.0 | 0.0 | 0.0 | 28.0 | 0.0 | 50.0 | 33.3 | 0.0 | 34.1 | 100.0 | 100.0 |  |
|  | Red | 66.7 | 0.0 | 17.9 | 0.0 | 0.0 | 0.0 | 0.0 | 38.2 | 0.0 | 0.0 | 0.0 |  |
|  | Transparent | 0.0 | 0.0 | 65.6 | 0.0 | 0.0 | 0.0 | 0.0 | 16.2 | 0.0 | 0.0 | 0.0 |  |
| Sediment | Blue | 13.7 | 5.8 | 0.0 | 69.4 | 0.0 | 0.0 | 0.0 | 17.5 | 25.0 | 0.0 | NA |  |
|  | White | 0.0 | 11.9 | 0.0 | 0.0 | 0.0 | 0.0 | 0.0 | 0.0 | 0.0 | 0.0 | NA |  |
|  | Gray | 0.0 | 12.5 | 0.0 | 0.0 | 0.0 | 0.0 | 0.0 | 0.0 | 23.4 | 0.0 | NA |  |
|  | Purple | 15.8 | 0.0 | 73.0 | 30.6 | 0.0 | 0.0 | 0.0 | 0.0 | 0.0 | 0.0 | NA |  |
|  | Black | 13.9 | 0.0 | 27.0 | 0.0 | 42.6 | 100.0 | 0.0 | 43.4 | 0.0 | 0.0 | NA |  |
|  | Red | 37.2 | 24.8 | 0.0 | 0.0 | 57.4 | 0.0 | 0.0 | 0.0 | 0.0 | 0.0 | NA |  |
|  | Transparent | 19.4 | 13.2 | 0.0 | 0.0 | 0.0 | 0.0 | 0.0 | 39.1 | 0.0 | 0.0 | NA |  |
|  | Green | 0.0 | 9.2 | 0.0 | 0.0 | 0.0 | 0.0 | 0.0 | 0.0 | 26.6 | 0.0 | NA |  |
|  | Brown | 0.0 | 22.7 | 0.0 | 0.0 | 0.0 | 0.0 | 0.0 | 0.0 | 25.0 | 0.0 | NA |  |
| SIZES | | | | | | | | | | | | | |
| Water | <1000 μm | 84.2 | 80.0 | 100.0 | 0.0 | 100.0 | 66.7 | 100.0 | 50.0 | 70.0 | 100.0 | NA |  |
|  | 1000-2000 μm | 0.0 | 0.0 | 0.0 | 100.0 | 0.0 | 33.3 | 0.0 | 37.5 | 0.0 | 0.0 | NA |  |
|  | 2000-3000 μm | 10.5 | 20.0 | 0.0 | 0.0 | 0.0 | 0.0 | 0.0 | 0.0 | 0.0 | 0.0 | NA |  |
|  | 3000-4000 μm | 5.3 | 0.0 | 0.0 | 0.0 | 0.0 | 0.0 | 0.0 | 12.5 | 30.0 | 0.0 | NA |  |
|  | >4000 μm | 0.0 | 0.0 | 0.0 | 0.0 | 0.0 | 0.0 | 0.0 | 0.0 | 0.0 | 0.0 | NA |  |
| Zooplankton | <1000 μm | 100.0 | 100.0 | 100.0 | 100.0 | 0.0 | 66.7 | 100.0 | 66.7 | 33.3 | 75.0 | 66.7 |  |
|  | 1000-2000 μm | 0.0 | 0.0 | 0.0 | 0.0 | 0.0 | 33.3 | 0.0 | 33.3 | 33.3 | 0.0 | 33.3 |  |
|  | 2000-3000 μm | 0.0 | 0.0 | 0.0 | 0.0 | 0.0 | 0.0 | 0.0 | 0.0 | 33.3 | 25.0 | 0.0 |  |
|  | 3000-4000 μm | 0.0 | 0.0 | 0.0 | 0.0 | 0.0 | 0.0 | 0.0 | 0.0 | 0.0 | 0.0 | 0.0 |  |
|  | >4000 μm | 0.0 | 0.0 | 0.0 | 0.0 | 0.0 | 0.0 | 0.0 | 0.0 | 0.0 | 0.0 | 0.0 |  |
| Sediment | <1000 μm | 0.0 | 51.4 | 60.0 | 57.1 | 22.2 | 12.5 | 0.0 | 87.5 | 33.3 | 0.0 | NA |  |
|  | 1000-2000 μm | 0.0 | 37.8 | 20.0 | 42.9 | 33.3 | 0.0 | 0.0 | 12.5 | 0.0 | 0.0 | NA |  |
|  | 2000-3000 μm | 20.0 | 2.7 | 20.0 | 0.0 | 11.1 | 12.5 | 0.0 | 0.0 | 33.3 | 0.0 | NA |  |
|  | 3000-4000 μm | 0.0 | 8.1 | 0.0 | 0.0 | 0.0 | 0.0 | 0.0 | 0.0 | 0.0 | 0.0 | NA |  |
|  | >4000 μm | 80.0 | 0.0 | 0.0 | 0.0 | 33.3 | 75.0 | 0.0 | 0.0 | 33.3 | 0.0 | NA |  |
| POLYMERS | | | | | | | | | | | | | |
| Water | Acrylic | 0.0 | 33.0 | 33.3 | 0.0 | 0.0 | 0.0 | 0.0 | 0.0 | 0.0 | 0.0 | NA |  |
|  | Nylon | 36.5 | 0.0 | 0.0 | 0.0 | 0.0 | 0.0 | 0.0 | 0.0 | 0.0 | 0.0 | NA |  |
|  | Polyethylene | 0.0 | 0.0 | 33.3 | 0.0 | 40.0 | 25.0 | 35.0 | 0.0 | 9.0 | 0.0 | NA |  |
|  | Polyester | 27.0 | 67.0 | 16.7 | 100.0 | 60.0 | 75.0 | 40.0 | 85.0 | 76.0 | 82.0 | NA |  |
|  | Polyestirene | 36.5 | 0.0 | 16.7 | 0.0 | 0.0 | 0.0 | 25.0 | 15.0 | 0.0 | 0.0 | NA |  |
|  | Rayon | 0.0 | 0.0 | 0.0 | 0.0 | 0.0 | 0.0 | 0.0 | 0.0 | 15.0 | 18.0 | NA |  |
| Zooplankton | Acrylic | 33.3 | 70.0 | 0.0 | 0.0 | 0.0 | 50.0 | 0.0 | 45.0 | 0.0 | 0.0 | 50 |  |
|  | Nylon | 0.0 | 0.0 | 0.0 | 80.0 | 0.0 | 0.0 | 0.0 | 0.0 | 0.0 | 65.0 | 0 |  |
|  | Polyester | 66.7 | 30.0 | 66.7 | 20.0 | 0.0 | 50.0 | 65.0 | 15.0 | 25.0 | 35.0 | 50 |  |
|  | Rayon | 0.0 | 0.0 | 33.3 | 0.0 | 0.0 | 0.0 | 35.0 | 0.0 | 75.0 | 0.0 | 0 |  |
|  | Polyamide | 0.0 | 0.0 | 0.0 | 0.0 | 0.0 | 0.0 | 0.0 | 40.0 | 0.0 | 0.0 | 0 |  |
| Sediment | Acrylic | 50.0 | 10.0 | 50.0 | 55.0 | 30.0 | 20.0 | 0.0 | 0.0 | 50.0 | 0.0 | NA |  |
|  | Polyester | 50.0 | 20.0 | 50.0 | 45.0 | 70.0 | 55.0 | 0.0 | 20.0 | 50.0 | 0.0 | NA |  |
|  | Rayon | 0.0 | 70.0 | 0.0 | 0.0 | 0.0 | 0.0 | 0.0 | 80.0 | 0.0 | 0.0 | NA |  |
|  | Polyamide | 0.0 | 0.0 | 0.0 | 0.0 | 0.0 | 25.0 | 0.0 | 0.0 | 0.0 | 0.0 | NA |  |

NA = Not processed due to the lack of sampling

**Table S2.** Microplastic concentration at each sampling site in the Sontecomapan lagoon in water (items/L), zooplankton (items/ind), and sediment (items/kg).

| Station | | 1 | 2 | 3 | 4 | 5 | 6 | 7 | 8 | 9 | 10 | 11 |
| --- | --- | --- | --- | --- | --- | --- | --- | --- | --- | --- | --- | --- |
| Water | | 19.00 | 5.00 | 6.00 | 1.00 | 5.00 | 9.00 | 4.00 | 7.00 | 12.00 | 7.00 | NA |
| Zooplankton | Copepods | 0.001 | 0.018 | 0.000 | 0.004 | 0.000 | 0.000 | 0.002 | 0.000 | 0.000 | 0.000 | 0.000 |
|  | Chaetognaths | NA | NA | 0.036 | NA | 0.000 | 0.010 | 0.000 | 0.020 | 0.010 | 0.010 | 0.000 |
|  | Luciferids | NA | 0.050 | 0.020 | NA | NA | 0.022 | 0.000 | 0.030 | 0.020 | 0.000 | 0.013 |
| Sediment | | 4.80 | 43.01 | 7.04 | 7.94 | 8.69 | 2.70 | 0.00 | 8.59 | 2.44 | 0.00 | NA |

NA = Not processed due to the lack of sampling or low number of animals
